# Linkage and association analyses of microsatellites and single-nucleotide polymorphisms in nuclear families

**DOI:** 10.1186/1471-2156-6-S1-S25

**Published:** 2005-12-30

**Authors:** Jennifer Lin, Kuang-Yu Liu

**Affiliations:** 1 Division of Preventive Medicine, Brigham and Women's Hospital, Harvard Medical School, Boston, MA 02215, USA; 2 HCNR Center for Bioinformatics, Harvard Medical School, Boston, MA 02115, USA

## Abstract

Several simulation studies have suggested that a high-density single-nucleotide polymorphisms (SNPs) marker set may be as useful as a traditional microsatellites (MS) marker set in performing whole-genome linkage analysis. However, very few studies have directly tested the SNPs-based genome-wide scan. In the present study, we compared the linkage results from the SNPs-based scan with a map density of 3-cM spacing with those from the MS scan using a 10-cM marker set among 300 nuclear families each from the Aipotu (AI), Danacaa (DA), and Karangar (KA) populations from the simulated Genetic Analysis Workshop 14 Problem 2 data. We found that information contents obtained from the SNPs scan were somewhat lower than those from the MS scan. However, the linkage results obtained from the two scans showed a high degree of similarity. Both scans identified a similar number of chromosomal regions attaining nominal significance (*p *< 0.05). Specifically, both scans detected confirmed evidence for linkage (NPL ≥ 4.07, *p *= 2 × 10^-5^) to chromosome 1 in the AI families, chromosomes 1 and 3 in the DA families, and chromosomes 3, 5, and 9 in the KA families. An additional confirmed linkage to chromosome 5 in the AI families was detected only by the MS scan. We also observed slightly wider 1-LOD intervals for more of the SNP peaks than for the MS peaks, which is likely due to lower information contents for the SNPs. Subsequent fine-mapping association analysis further identified 2 to 3 markers significantly associated with disease status in each population; B03T3056, B03T3058, and B05T4139 in the AI population, B03T3056 and B03T3058 in the KA population, and B03T3056, B03T3057, and B03T3058 in the DA population. Among the four markers, three were chosen based on results obtained from the two scans, but one was solely from the SNP scan. In summary, our finding suggests that the SNP-based genome scan has the potential to be as powerful as the traditional MS-based scan and offers good identification of peak location for further fine-mapped association analysis.

## Background

Relative to microsatellites (MS), single-nucleotide polymorphisms (SNPs) are more abundantly and uniformly distributed along the human genome, and they are more reliably typed and require a smaller DNA sample [1]. Using SNPs to perform a linkage-based genome-wide scan becomes possible as lower-cost, high-throughput SNP genotyping is made available [1]. Although diallelic SNPs offer less information (lower heterozygosity) than multialleic MS, it has been suggested by several simulation studies that an increase in SNP map density would compensate for the lower information content [2-4].

To learn whether a SNP marker set could be as useful as the standard MS marker set for the linkage-based genome scan, we compared both MS- and SNPs-based genome-wide scans among nuclear families in the three populations using the simulated Genetic Analysis Workshop 14 (GAW14) Problem 2 data. Subsequent fine-mapped analyses were performed in regions showing `confirmed' evidence for linkage.

## Methods

We pooled together the last 3 replicates (98–100), which provided us with 300 nuclear families each from the Aipotu (AI), Danacaa (DA), and Karangar (KA) populations. All analyses were performed without knowledge of the answers.

Hardy-Weinberg (HW) tests were first carried out for the 917 SNPs using founder genotypes in each population. Seventeen, 16, and 20 SNPs in the AI, DA, and KA populations were not in HW equilibrium and were dropped from further analysis. Two genome-wide scans with 416 MS (10-cM intermarker spacing) and ~900 SNPs (3-cM intermarker spacing) were then performed among families from the three populations. All SNP pairs were not in strong linkage disequilibrium (LD, r^2 ^< 0.7). We used a multipoint nonparametric linkage (NPL) scoring method as implemented in the program GENEHUNTER [5] to assess evidence for linkage. Allele frequencies were based on those supplied by the map files, and all the linkage analyses used the "ALL" scoring statistic. The scanning was performed at each marker, with no estimates between markers.

Subsequent fine-mapped association analysis was carried out by comparing the distribution of single-marker alleles and two-marker haplotypes between affected offspring from the three populations and unrelated controls. The frequencies of single marker alleles and two-marker haplotypes within each population were estimated by an expectation maximization (EM) algorithm implemented in the TRANSMIT program [6]. Test for difference in allele or haplotype distribution between affected offspring and controls was a generalized likelihood ratio test, 2x|*ln*(*L*_*affectedoffspring*_) + *ln*(*L*_*controls*_) - *ln*(*L*_*combined*_))|, where *L *is the estimate of maximum likelihood for haplotype frequency. This has asymptotically a χ^2 ^distribution with (number of alleles or number of haplotypes -1) degrees of freedom under the null hypothesis. Test for transmission of a two-marker haplotype from parents to an affected offspring employed a score test implemented in the TRANSMIT program [6]. The degree of linkage LD between marker pairs was estimated with the *r*^2 ^statistic on the basis of founder genotypes with an EM algorithm implemented in the GOLD-LDMAX program [7]. Multiple test comparison was corrected with the false-discovery rate (FDR) correction implemented in the Q-VALUE program [8].

## Results

### Genome-scan linkage analyses

We observed that information contents (ICs) were lower in the SNP scan relative to those in the MS; the average ICs across the genome for the SNPs was ~10% lower (82% in the AI, DA, and KA families) than that for the MS (93% for the AI and KA families and 92% for DA families). However, there was good concordance of results between the MS and SNP scans; both scans identified a similar number of chromosomal regions attaining nominal significance for linkage (NPL > 1.65, *p *< 0.05) at two or more adjacent markers. Specifically, both scans detected 8, 5, and 8 chromosomal regions in the AI, DA, and KA families, respectively (Table 1). Four linkage signals were detected only by either scan (two each by either scan) in the AI and DA families (Table 1). Moreover, both scans identified several "confirmed" linkage evidence (NPL ≥ 4.07, *p *= 2 × 10^-5^) (Table 1). In the AI families, both scans detected one confirmed linkage to chromosome 3 with a NPL of 6.6 at MS marker D03S0127 (MS) and a NPL of 5.5 at SNP marker C03R0280. In the DA families, confirmed linkage evidence was detected on chromosome 1, with a NPL of 7.6 at MS marker D01S0024 and a NPL of 8.6 at SNP marker C01R0052, and also on chromosome 3 with a NPL of 5.2 at MS marker D03S0127 and a NPL of 4.5 at SNP marker C03R0281. In the KA families, confirmed linkage evidence was observed on chromosomes 3 with a NPL of 6.1 at MS marker D03S0127 and a NPL of 6.3 at SNP marker C03R0281, on chromosome 5 with a NPL of 7.5 at MS marker D05S0173 and a NPL of 7.2 at SNP marker C05R0380, and on chromosome 9 with a NPL of 7.2 at MS marker D09S0437 and a NPL of 6.3 at SNP marker C09R0765. The only exception is that one confirmed linkage to chromosome 5 was detected only by the MS scan in the AI families with a NPL of 4.5 at MS marker D05S0173.

When comparing linkage peaks obtained from the two scans, we found that the peak locations identified by the two scans were mostly close. The average distance between the MS- and SNP-peak locations ranged from 0.05 to 10 cM, likely representing gaps of spacing in the two maps. When examining the 1-LOD support interval of linkage peaks using a 1 cM-increment map, we found that 1-LOD supportive interval of these peaks by the two scans covered comparable regions. However, there were exceptions, including the peaks on chromosome 1 from the KA families (43 cM between the MS peak D01S0018 and SNP peak C01R0053), on chromosome 6 from the AI families (22 cM between the peaks-D06S0229 and C06R0510), and on chromosome 7 from the DA families (60 cM between the peaks-D07S0272 and C07R00623), suggesting that the two scans detected different signals in these chromosomal regions. Additionally, most of the MS peaks covered slightly narrower 1-LOD intervals relative to the SNP peaks (Table 1). Two SNP-peaks covering 20 cM more than the MS-peaks were on chromosome 8 from the AI families and on chromosome 3 from the DA families (Table 1). On the contrary, the SNP-peaks on chromosome 1 from the AI and DA families and on chromosomes 5 and 9 from the KA families had slightly narrower 1-LOD intervals than the corresponding MS-peaks.

### Fine-mapped analyses

In the follow-up analysis, we chose to focus on the markers showing confirmed evidence for linkage (NPL ≥ 4.07) in the two scans in any of the three populations. This resulted in 3 (D03S0127, C03R0280, D05S0173), 4 (D01S0024, C01R0052, D03S0127, C03R0281), and 6 (D03S0127, C03R0279, D05S0173, C05R0380, D09S0347, C09R0765) candidate markers from the AI, DA, and KA families, respectively. Accordingly, we acquired 6 20-marker data from packets 28, 29, 153, 207, 208, and 417, with genotypes for the three populations and one control sample. Each packet covers an average spacing of 5 cM. Packets 29, 153, 208, and 417 covered the linkage signals detected by the MS scan, and packets 28, 153, 207, and 417, by the SNP scan.

We repeated linkage analysis on chromosomes 1, 3, 5, and 9 with addition of the acquired markers. An increase in the ICs and NPL scores of the original peaks were observed, a clear confirmation of the original evidence for linkage (Table 1). Specifically, the NPL scores over the fine-mapped region were ~10% higher than the original scores. Slight shift of locations for some peaks were also observed, likely due to difference in the map density between both of the fine-mapped and original scans.

### Linkage disequilibrium (LD), allele association, and transmission disequilibrium tests

Pairwise LD for the acquired SNPs among the three populations was not strong; the *r*^2 ^for majority of the SNP pairs was less than <0.7 in all packets. The only exception was the marker pair, B09T8338 and B09T8339; the *r*^2 ^value was around 0.9 for all the three populations. However, similar magnitude of LD for that pair was also observed in the controls.

We then examined single-marker allelic distribution between the affected offspring from the three populations and controls at the 120 acquired markers. After adjusting for multiple testing for an FDR level of 0.05, we found that B05T4139 from packet 207 was significantly associated with affection status in the AI population and that the marker was acquired by the SNP scan. Additionally, 3 consecutive markers (B03T3056, B03T3057, B03T3058) from packet 153 were significant in the DA population and 2 markers (B03T3056 and B03T3058) were significant in the AI and KA populations. Table 2 provides the maximum likelihood estimate of the risk allele frequency of these markers in the affected offspring and controls.

We extended two-marker haplotype association on the two markers, B03T3056 and B03T3058, which showed significant single-marker association in the three populations. Using a likelihood ratio test, we further observed significant difference in haplotype distribution between the affected offspring and controls for the marker pair (Table 3). Specifically, haplotype 11 (i.e., allele 1 for marker B03T3056 combined with allele 1 for marker B03T3058) was significantly over-transmitted from parents to offspring in the three populations (Table 3).

## Discussion

In the present study, we observed a high degree of correspondence of results from the MS and SNP genome scans. Although lower ICs were present for the SNPs relative to those for the MS, both the MS and SNP scans detected similar number of signals attaining nominal significance. Specifically, the two scans detected confirmed evidence for linkage to chromosome 1 in the AI families, chromosomes 1 and 3 in the DA families, and chromosomes 3, 5, and 9 in the KA families. However, one confirmed linkage to chromosome 5 in the AI families was detected only by the MS scan. The peak locations obtained from the two scans were mostly close. Moreover, we observed somewhat wider 1-LOD peak intervals for most of the SNPs relative to those for the MS, likely due to the lower ICs for the SNPs. Subsequent fine-mapped linkage analysis confirmed the initial linkage results. We also observed significant associations of the 4 markers with affection status, all of which could be acquired from the SNP scan. One haplotype from two of these markers was shown to be over-transmitted from parents to offspring in the three populations.

Two studies comparing a high-density SNP scan with a traditional MS scan have also shown a remarkable similarity of results from the two scans [9,10]. These two studies found that the SNPs with a high-density map (<0.2-cM spacing) provide substantially higher ICs, more linkage signals, and narrower linkage intervals than the MS [9,10]. In our study, the slightly lower IC and wider linkage intervals for the SNPs relative to the MS is likely attributable to the less dense map for the SNPs (i.e., 3 cM for intermarker spacing). Indeed, John and colleagues indicated that a reduction of the density of SNP set to one SNP per cM generated results that more closely resembled the MS scan. Findings from our study and from John et al. suggest that a denser SNP map (e.g., ≤ 1-cM spacing) may be necessary to ensure higher information contents.

In summary, our findings suggest that the SNP-based genome-scan has the potential to be as powerful as the traditional MS-based scan. In the present study, an average intermarker spacing of 3 cM offers comparable linkage results from the nuclear families to those based on the MS scan with an average spacing of 10 cM. In addition, fine-mapped association results further confirmed the utility of SNPs with good identification of peak locations. With the availability of a dense map, accurate map position, and low-cost high-throughput genotyping, we anticipate that SNPs may soon become useful in conducting both linkage and association studies.

## Abbreviations

EM: Expectation maximization

FDR: False-discovery rate

HW: Hardy-Weinberg

IC: Information content

LD: Linkage disequilibrium

MS: Microsatellites

NPL: Nonparametric linkage

SNP: Single-nucleotide polymorphism

## Authors' contributions

Both authors contributed to conception, analysis, and interpretation of data. JL drafted the article. Both authors read and approved the version for publication.

## Figures and Tables

**Table 1:** NPL*, IC*, and 1-LOD linkage interval for chromosomal regions attaining nominal significance and fine-mapped linkage results.

Pop.	Chr.	Scan	Marker	Position (cM)	NPL^a^	IC^a^	1-LOD interval
NPL, IC, and 1-LOD linkage intervals							
AI	1	MS^a^	D01S0025	181.59	3.64	0.93	62
	1	SNP^a^	C01R0053	172.08	3.84	0.86	51
	3	MS	D03S0127	295.84	6.6	0.92	22
	3	SNP	C03R0280	295.89	5.53	0.8	26
	4	MS	D04S0158	210.27	2.71	0.93	--
	4	SNP	C04R0349	212.91	1.78	0.83	--
	5	MS	D05S0173	9.69	4.53	0.94	23
	5	SNP	C05R0379	3.07	3.71	0.7	30
	6	MS	D06S0229	90.19	1.98	0.92	--
	6	SNP	C06R0510	112.24	2.09	0.83	--
	7^b^	SNP	C07R0666	299.48	1.77	0.73	--
	8	MS	D08S0328	175.32	2.49	0.92	137
	8	SNP	C08R0721	172.14	2.43	0.86	160
	9	MS	D09S0348	10.28	3.92	0.91	50
	9	SNP	C09R0770	20.28	3.28	0.76	52
							
DA	1	MS	D01S0024	173.88	7.63	0.93	33
	1	SNP	C01R0052	169.6	8.55	0.88	23
	3	MS	D03S0127	295.84	5.24	0.92	7
	3	SNP	C03R0281	298.31	4.5	0.79	33
	5^b^	MS	D05S0187	107.45	2.17	0.95	114
	6^b^	SNP	C06R0506	95.16	2.61	0.83	60
	7	MS	D07S0272	86.83	2.05	0.94	--
	7	SNP	C07R0623	160.4	2.08	0.86	--
	8	MS	D08S0341	265.38	3.11	0.91	47
	8	SNP	C08R0749	261.07	1.89	0.82	--
	9^b^	MS	D09S0348	10.28	1.81	0.89	--
	10	MS	D10S0406	115.16	2.91	0.94	74
	10	SNP	C10R0893	114.09	2.66	0.82	84
							
KA	1	MS	D01S0018	129.21	1.92	0.95	--
	1	SNP	C0140053	172.08	2.01	0.87	--
	2	MS	D02S0051	67.03	3.06	0.94	26
	2	SNP	C02R0113	68.97	2.90	0.83	37
	3	MS	D03S0127	295.84	6.12	0.92	12
	3	SNP	C03R0281	298.31	6.34	0.84	15
	4	MS	D04S0158	210.27	2.08	0.93	--
	4	SNP	C04R0348	209.3	1.77	0.86	--
	5	MS	D05S0173	9.69	7.49	0.95	21
	5	SNP	C05R0380	5.74	7.15	0.85	21
	9	MS	D09S0347	2.77	7.19	0.92	23
	9	SNP	C09R0765	5.83	6.3	0.78	23

Pop.	Chr.	Scan	Marker	Position (cM)	NPL	IC	1-LOD interval

Fine-mapped linkage results							
AI	3	153^c ^+ MS + SNP	B03T3055	297.14	7.4	0.99	
	5	208^c ^+ MS + SNP	B05T4142	7.57	5.71	0.97	
DA	1	28^c ^+ 29^c ^+ MS + SNP	B01T0561	169.7	9.46	0.98	
	3	153^c ^+ MS+ SNP	B03T3057	297.71	6.46	0.99	
KA	3	153^c ^+ MS+ SNP	C03R0281	298.31	7.38	0.97	
	5	207^c ^+ 208^c ^+ MS + SNP	B05T4136	5.22	8.87	0.99	
	9	417^c ^+ MS + SNP	B09T8337	5.98	8.36	0.96	

^a^NPL, Maximum nonparametric linkage scores; IC, information content; MS, microsatellites; SNP, single-nucleotide polymorphisms.

^b^Only results from one scan was reported because the other scan did not reach nominal significance.

^c^Packet number

**Table 2:** Allele frequency of the significant markers among affected offspring from the AI, DA, and KA families and unrelated controls.

Chr	Marker name	Position (cM)	Allele^a^	AI	DA	KA	Control
3	B03T3056	6.56	2	0.38	0.36	0.41	0.48
3	B03T3057	297.74	2	0.35^b^	0.37	0.33^b^	0.27
3	B03T3058	297.71	2	0.39	0.43	0.38	0.48
5	B05T4139	297.55	2	0.25	0.21^b^	0.22^b^	0.16

^a^Allele with rare frequency

^b^Marker was not significantly associated with disease after FDR correction.

**Table 3:** Associated haplotypes with affection status at a marker pair among affected offspring from the AI, DA, and KA families.

Chr	Marker pair	Population	Haplotype	Frequency	χ^2^	*p*-Value^a^	Odds^b^
3	B03T3056 &	AI	11	0.43	87.21	<0.0001	2.2
	B03T3058	DA	11	0.41	71.45	<0.0001	2.3
		KA	11	0.44	118.25	<0.0001	2.0

^a^Nominal *p*-value

^b^Ratio of frequency of one specific haplotype over that of the rest haplotypes

## References

[B1] Matise TC, Sachidanandam R, Clark AG, Kruglyak L, Wijsman E, Kakol J, Buyske S, Chui B, Cohen P, de Toma C, Ehm M, Glanowski S, He C, Heil J, Markianos K, McMullen I, Pericak-Vance MA, Silbergleit A, Stein L, Wagner M, Wilson AF, Winick JD, Winn-Deen ES, Yamashiro CT, Cann HM, Lai E, Holden AL (2003). A 3.9-centimorgan-resolution human single-nucleotide polymorphism linkage map and screening set. Am J Hum Genet.

[B2] Kruglyak L (1997). The use of a genetic map of biallelic markers in linkage studies. Nat Genet.

[B3] Kennedy GC, Matsuzaki H, Dong S, Liu WM, Huang J, Liu G, Su X, Cao M, Chen W, Zhang J, Liu W, Yang G, Di X, Ryder T, He Z, Surti U, Phillips MS, Boyce-Jacino MT, Fodor SP, Jones KW (2003). Large-scale genotyping of complex DNA. Nat Biotechnol.

[B4] Wilson AF, Sorant AJ (2000). Equivalence of single- and multilocus markers: power to detect linkage with composite markers derived from biallelic loci. Am J Hum Genet.

[B5] Kruglyak L, Daly MJ, Reeve-Daly MP, Lander ES (1996). Parametric and nonparametric linkage analysis: a unified multipoint approach. Am J Hum Genet.

[B6] Clayton D (1999). A generalization of the transmission/disequilibrium test for uncertain-haplotype transmission. Am J Hum Genet.

[B7] Abecasis GR, Cookson WO (2000). GOLD – graphical overview of linkage disequilibrium. Bioinformatics.

[B8] Storey JD, Tibshirani R (2003). Statistical significance for genomewide studies. Proc Natl Acad Sci U S A.

[B9] Middleton FA, Pato MT, Gentile KL, Morley CP, Zhao X, Eisener AF, Brown A, Petryshen TL, Kirby AN, Medeiros H, Carvalho C, Macedo A, Dourado A, Coelho I, Valente J, Soares MJ, Ferreira CP, Lei M, Azevedo MH, Kennedy JL, Daly MJ, Sklar P, Pato CN (2004). Genomewide linkage analysis of bipolar disorder by use of a high-density single-nucleotide-polymorphism (SNP) genotyping assay: a comparison with microsatellite marker assays and finding of significant linkage to chromosome 6q22. Am J Hum Genet.

[B10] John S, Shephard N, Liu G, Zeggini E, Cao M, Chen W, Vasavda N, Mills T, Barton A, Hinks A, Eyre S, Jones KW, Ollier W, Silman A, Gibson N, Worthington J, Kennedy GC (2004). Whole-genome scan, in a complex disease, using 11,245 single-nucleotide polymorphisms: comparison with microsatellites. Am J Hum Genet.

